# A machine learning model to predict heart failure readmission: toward optimal feature set

**DOI:** 10.3389/frai.2024.1363226

**Published:** 2024-02-21

**Authors:** Sonia Jahangiri, Masoud Abdollahi, Ehsan Rashedi, Nasibeh Azadeh-Fard

**Affiliations:** Industrial and Systems Engineering Department, Rochester Institute of Technology, Rochester, NY, United States

**Keywords:** readmission, heart failure, machine learning, feature selection, clinical decision making

## Abstract

**Background:**

Hospital readmissions for heart failure patients remain high despite efforts to reduce them. Predictive modeling using big data provides opportunities to identify high-risk patients and inform care management. However, large datasets can constrain performance.

**Objective:**

This study aimed to develop a machine learning based prediction model leveraging a nationwide hospitalization database to predict 30-day heart failure readmissions. Another objective of this study is to find the optimal feature set that leads to the highest AUC value in the prediction model.

**Material and methods:**

Heart failure patient data was extracted from the 2020 Nationwide Readmissions Database. A heuristic feature selection process incrementally incorporated predictors into logistic regression and random forest models, which yields a maximum increase in the AUC metric. Discrimination was evaluated through accuracy, sensitivity, specificity and AUC.

**Results:**

A total of 566,019 discharges with heart failure diagnosis were recognized. Readmission rate was 8.9% for same-cause and 20.6% for all-cause diagnoses. Random forest outperformed logistic regression, achieving AUCs of 0.607 and 0.576 for same-cause and all-cause readmissions respectively. Heuristic feature selection resulted in the identification of optimal feature sets including 20 and 22 variables from a pool of 30 and 31 features for the same-cause and all-cause datasets. Key predictors included age, payment method, chronic kidney disease, disposition status, number of ICD-10-CM diagnoses, and post-care encounters.

**Conclusion:**

The proposed model attained discrimination comparable to prior analyses that used smaller datasets. However, reducing the sample enhanced performance, indicating big data complexity. Improved techniques like heuristic feature selection enabled effective leveraging of the nationwide data. This study provides meaningful insights into predictive modeling methodologies and influential features for forecasting heart failure readmissions.

## 1 Introduction

Hospital readmission is considered an accountability measure and quality indicator for healthcare in the United States (Low et al., 2015). The Centers for Medicare and Medicaid Services (CMS) implemented the Hospital Readmissions Reduction Program (HRRP) in October 2012 as part of the Affordable Care Act (ACA) (Qiu et al., 2022). This program mandates CMS to adjust hospital reimbursements based on their readmission rates (Qiu et al., 2022). Focusing on six specific medical conditions, including heart failure (HF), myocardial infarction (MI), Chronic obstructive pulmonary disease (COPD), Coronary artery bypass graft (CABG) surgery, total hip/knee arthroplasty (THA/TKA), and pneumonia, CMS has initiated public reporting of 30-day risk-standardized readmission (National Quality Form, 2008; CMS, 2023). HF affects over 26 million individuals globally, resulting in more than one million hospitalizations annually in the United States (Sarijaloo et al., 2021). The prevalence of HF is steadily increasing due to the aging population. Data from 2015 to 2018 shows ~6 million American adults aged 20 years and above were diagnosed with HF (Virani et al., 2021). Forecasts indicate that this number is expected to surge to eight million by 2030, leading to associated costs of $55 billion (Savarese and Lund, 2017). Post-discharge readmission or mortality poses significant challenges to healthcare for patients with HF. Within 30 days of discharge, up to 25% of HF patients may face readmission, with an associated mortality risk of ~10% (Krumholz et al., 2009). Despite nationwide efforts focused on decreasing readmission rates for HF exacerbations, evidence indicates that 30-day readmission and mortality for these patients are still rising (Gupta et al., 2018).

Data plays a crucial role in healthcare extracting invaluable knowledge and insights (Auffray et al., 2016). The abundance of patient information collected from diverse sources has given rise to data analytics as a powerful tool for comprehending intricate medical conditions (Shameer et al., 2017; Jahangiri et al., 2024). Given the significance of reducing readmission rates, numerous studies have been conducted to explore the factors influencing readmission rates among HF patients. For instance, in a recent study by Sharma et al., an HF readmission prediction model (a tree-based classifier) was developed using factors like sex, age, emergency department visits, and so on, achieving a c-statistics of 0.65 (Sharma et al., 2022). Similarly, Mortazavi et al. devised a random forest (RF) model that incorporated the aforementioned factors, comorbidity, race, and severity index, resulting in a c-statistics of 0.62 and a precision of 0.32 (Mortazavi et al., 2016). Several other studies on the same subject (Philbin and DiSalvo, 1999; Ross et al., 2008; Awan et al., 2019a,b) yielded performance levels of < 0.66. Furthermore, there is a rising trend in the number of studies around this topic recently. For instance, within the last 12 months, there were several studies employing machine learning (ML) methods to forecast the risk of readmission for patients with HF (Ru et al., 2023; Tong et al., 2023; Scholten et al., 2024). C-statistics of the developed models in these studies were in the range of 0.59–0.63. However, most of these studies have been limited by their use of small datasets with < 50,000 samples, potentially impeding the generalizability of their findings. To address this gap in the existing literature, it is essential to employ larger datasets collected at a national level. The Nationwide Readmissions Database (NRD) stands out as one of the most suitable datasets, encompassing nationwide data, and its recent sample size for HF patients in 2020 exceeds 500,000 discharge records.

The variability in studies that predict heart failure readmission (HFR) can be attributed to several factors, including the selection of features used in the predictive models. A thorough literature analysis reveals over 150 potential features that could be considered when developing a predictive model for HFR. These features can be broadly categorized into five classes: (1) demographics and socioeconomics, (2) clinical information or discharge information, (3) hospital-related information, (4) comorbidities, and (5) diagnosis and procedure-related information. Notably, the literature shows that researchers commonly implemented features from the demographics, clinical information, and comorbidities classes in their analysis (Guo et al., 2020; Rahman et al., 2023). However, studies such as Golas et al. (2018) and Ashfaq et al. (2019) have highlighted the significance of previously underexplored predictors as diagnosis and procedure-related information in predicting HFR. Surprisingly, these factors received little attention in earlier studies like (Zheng et al., 2015; Mortazavi et al., 2016; Awan et al., 2019b; Sharma et al., 2022). Consequently, it remains to be investigated whether considering these factors alongside others on a large dataset will lead to their identification as significant predictors. Further research is warranted to explore their potential impact on HFR prediction.

To bridge the mentioned gaps in the literature, this study aims to develop a machine learning-based HFR prediction model that uses a nationwide dataset. The dataset is rich in the number of records and encompasses a broad spectrum of distinct feature collections. One particularly notable feature collection within this dataset relates to diagnostic attributes. Another objective examined in this study involves employing an innovative process for selecting significant features in conjunction with ML techniques. This method's primary purpose is to assess each feature's potential influence on the prediction model by progressively integrating them into the training process. This technique facilitates the identification of feature combinations that yield high-performance metrics. A predictive model that emphasizes such a heuristic method could lead us to recognize the optimal feature set in HFR prediction. This study also compared the implementation of three distinct techniques for addressing imbalanced data challenges, along with utilizing the feature normalization method for each ML approach. Ultimately, these mentioned elements result in creating a prediction model characterized by an optimal selection of features with a notable Area Under the Receiver Operating Characteristic Curve (AUC) value.

## 2 Materials and methods

### 2.1 The dataset

The NRD is a unique and powerful readmission analysis database, produced by the Healthcare Cost and Utilization Project (HCUP). The NRD effectively fills a substantial gap in healthcare data by providing comprehensive and nationally representative information on hospital readmissions for all patients, irrespective of their expected payer for the hospital stay. The primary objective behind the establishment of such comprehensive data is to enhance national readmission analyses and offer invaluable support to health professionals, administrators, policymakers, and clinicians in their decision-making processes (Agency for Healthcare Research Quality, 2020).

**Figure 1:**
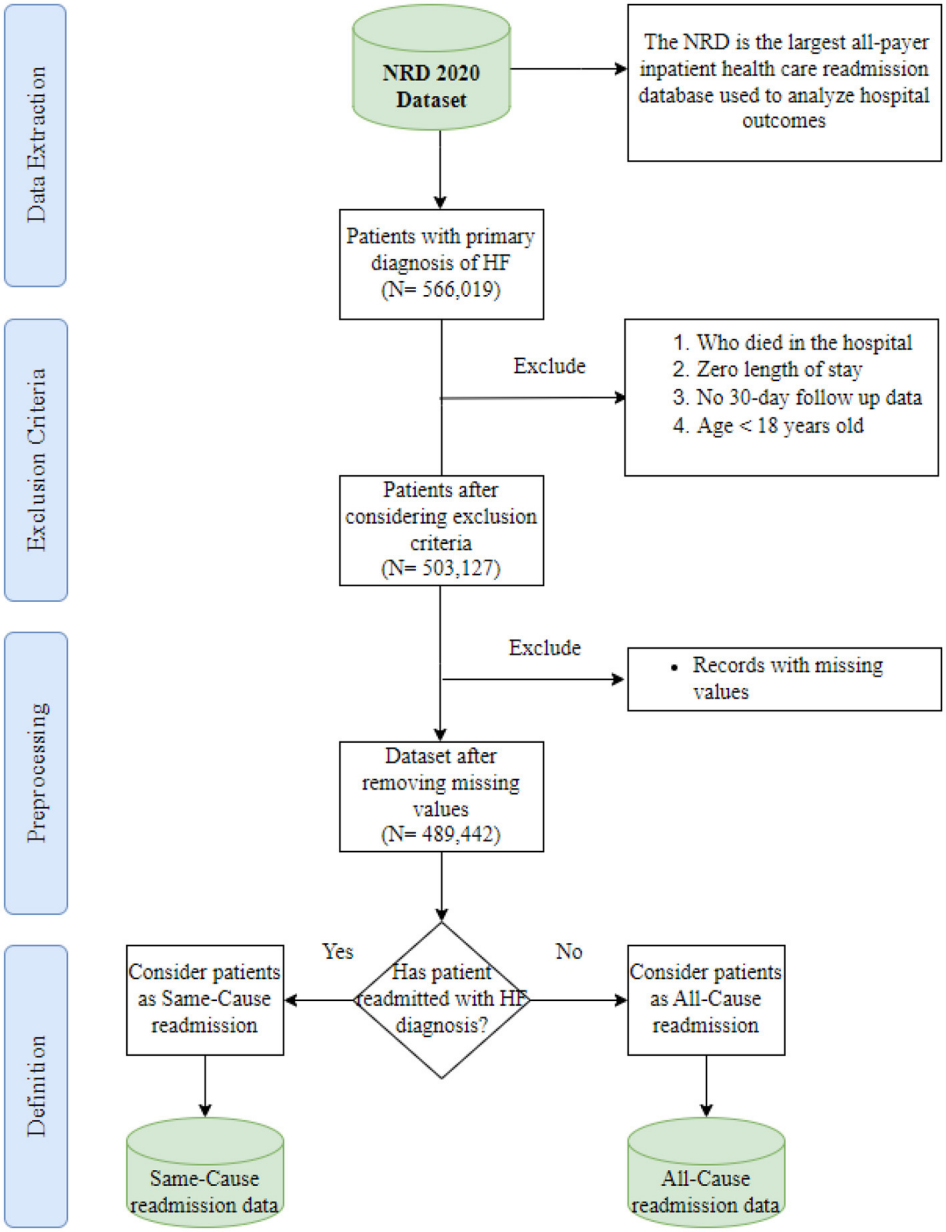
Overview of the data extraction process.

The study utilized the 2020 NRD dataset, which comprises discharge-level hospitalization data from 31 geographically diverse states, representing ~62.2% of the total U.S. resident population and about 60.8% of all hospitalizations during the specified period. This extensive dataset contains a significant volume of discharges, estimated to be around 16 million (weighted estimate of ~32 million discharges). The NRD dataset includes the International Classification of Diseases, Tenth Revision (ICD-10) codes. These codes constitute a comprehensive classification system incorporating various medical conditions, diseases, symptoms, injuries, and related health issues (CDC, 2015). In this paper, we extracted all hospital admissions with ICD-10 codes related to HF and stored patient information separately. After initial identification, 566,019 discharges with early HF diagnoses were found, and specific exclusions were applied, including cases with zero length of stay, in-hospital mortalities, lack of 30-day follow-up data (patients discharged in December 2020), and patients under 18 years old. Subsequently, records with missing values were removed, resulting in a final dataset comprising 489,442 records for analysis. Figure 1 depicts the study cohort design derived from the 2020 NRD data, which serves as the basis for analysis in our research.

### 2.2 Data analysis

#### 2.2.1 Data preparation

In the NRD, patients can be monitored over a year using a unique linkage number. To calculate the time to readmission, we computed the interval between two admissions, then subtracted the length of stay for the initial admission from the interval. The evaluation in this paper specifically concentrated on 30-day readmissions, extracting two types of readmissions from the dataset. The first type included all-cause readmissions, containing readmissions related to HF diagnosis and other diagnoses. The second type focused on same-cause readmissions, involving readmissions directly attributed to HF (With the same ICD-10 codes). The 2020 NRD database comprises discharge-level files and patient demographics, including age, expected primary payer, discharge status, and total charges. It also includes severity-related data for assessing the patient's condition, hospital-related information such as bed size and teaching status, and comorbidities like drug abuse and diabetes. Moreover, it contains diagnosis and procedure-related information, providing additional details through ICD-10-CM diagnoses and ICD-10-PCS procedures generated by HCUP software tools. Considering all this information, the initial dataset resulted in over 600 features.

We employed two methods for categorical and continuous variables to simplify the prediction model and enhance analysis speed to select the most important ones. Firstly, we used contingency tables to explore the relationship between categorical variables and the readmission rate. Secondly, the Logistic platform was utilized to fit a logistic regression model to analyze readmission rates while considering continuous variables. The analysis results included contingency tables with frequency counts and proportions, chi-square tests and *p*-values to assess significance. Through these tests, we selected the most significant features (considering *p*-values < 0.01) for predicting readmission, resulting in 31 features for the same-cause dataset and 30 for the all-cause dataset. This process helped streamline the model and improve the efficiency of our predictive analysis.

#### 2.2.2 Feature selection

To identify features leading to a high AUC in the readmission prediction model, a heuristic feature selection approach marked by a systematic, multistep procedure was employed. In the initial step, the model was constructed with the inclusion of just one feature, and its performance was assessed. This step generated 31 models for the same-cause dataset and 30 models for the all-cause dataset, each focusing on a singular feature. Subsequently, the AUC, a widely acknowledged metric in readmission analysis (Guo et al., 2020), was calculated for each model, establishing a baseline for individual feature performance.

Moving forward, a dynamic feature set was formulated, condensing the feature that demonstrated the highest AUC value among the single-feature models. The process then transitioned to evaluating the performance of the model by introducing combinations of the selected features with each remaining feature, systematically calculating the AUC value for each pair. This iterative exploration enabled the continual refinement of the feature set, identifying pairs with the highest AUC at each step. The process continued, analyzing the fluctuation of AUC in the HFR prediction model with the progressive addition of each feature. The final model selected, with the maximum AUC, served as the baseline model for subsequent analyses. This iterative approach systematically selected the most relevant feature sets, unraveling the nuanced pattern of performance changes with the inclusion of each feature. The goal of this process is to create a sophisticated and refined predictive model designed for readmission analysis.

#### 2.2.3 Machine learning models

Only a minority of patients, specifically 8.9 and 20.6%, experienced readmission to the hospital due to same-cause and all-cause conditions, respectively. These relatively low percentages highlight a common issue in medical datasets—imbalanced data. Such datasets often show a considerable disparity between the occurrence of outcome events and the cases where outcomes do not occur. Three distinct techniques, under-sampling, over-sampling, and SMOTE, were applied to address this issue within our dataset. Subsequently, a comparative analysis of the effectiveness of each ML method using these techniques was conducted. Implementing methods to address class imbalance increases the prediction model's sensitivity, thus improving its capacity to recognize potential outcomes. This means the model becomes more adept at correctly identifying instances that might lead to certain results, contributing to better overall performance.

Following the feature selection phase that identified the most critical attributes, two ML techniques—Logistic Regression (LR) and Random Forest (RF)—were employed on both same-cause and all-cause datasets. LR is a frequently employed technique in the existing literature for examining readmission (Artetxe et al., 2018). At the same time, the selection of RF is attributed to the strong performance of tree-based methods in predicting readmission, as documented in previous studies (Shams et al., 2015; Mortazavi et al., 2016). To ensure unbiased assessment, the dataset was partitioned into three segments: a training set, validation set, and test set, divided randomly in a 70:15:15 ratio. The training set facilitated model training, the validation set enabled hyperparameter exploration (e.g., number of trees in the forest in RF, or algorithm to use in the optimization problem in LR), and the test set enabled performance comparison of the proposed approach. In implementing the ML methods, feature normalization using standardization was adopted. Both normalized and unnormalized features were subjected to each algorithm to evaluate their performance. Consequently, the study involves two ML techniques, three techniques for handling class imbalance, and two states regarding feature normalization—resulting in 12 distinct conditions for model implementation. Ultimately, each condition's discriminatory ability was compared by calculating accuracy, sensitivity, specificity, and AUC.

### 2.3 Software packages

The dataset was loaded, and HF-related information was extracted using SPSS software version 28.0. Significant feature selection underwent statistical analysis utilizing JMP software version 16.1. The implementation code for ML methods, incorporating sequential feature selection, was scripted in Python programming environment version 3.9. The execution of Python code was carried out in the Visual Studio coding environment. The Python packages employed in our implementation include Pandas, NumPy, Scikit-learn (Sklearn), Statistics, and Imbalanced-learn (Imblearn). These packages collectively facilitated the implementation of ML algorithms and the execution of the feature selection process. Specifically, Scikit-learn was utilized for logistic regression and random forest models.

## 3 Results

### 3.1 Study characteristics

During the study period from January to November 2020, 566,019 discharges with primary HF diagnoses were recorded using ICD-10 codes. After applying exclusion criteria and addressing missing values, the dataset was refined to a final count of 489,442 records. The code “NRD_VisitLinks” was utilized to track patients with multiple admissions uniquely, and then data was divided into two sets based on whether their readmissions were due to the same cause or any cause. The baseline characteristics of patients in the 30-day analytical samples for both same-cause and all-cause datasets are presented in Table 1.

**Table 1:** Baseline characteristics for some of the features, (mean ± SD) for continuous variables; n (%) for categorical variables.

**Features**	**Same-cause 30-day readmission**		**All-cause 30-day readmission**	
	**Yes**	**No**	**Yes**	**No**
Total	8.91%	91.09%	20.65%	79.35%
**Demographic characteristics**				
Age (years)	68.5 ± 14.6	71.7 ± 14	70.1 ± 14.1	71.7 ± 14.1
LOS (days)	5.4 ± 5.1	5.6 ± 6.1	5.9 ± 6.6	5.5 ± 5.9
Gender (female)	42.10%	47.20%	45.16%	47.10%
**Payment method**				
Medicare	68.19%	72.50%	71.85%	72.20%
Medicaid	18.74%	11.40%	15.21%	11.20%
Private insurance	8.13%	10.90%	8.60%	11.20%
Self-pay	2.40%	2.50%	2%	2.70%
Other	2.54%	2.70%	2.34%	2.70%
**Disposition of patient**				
Routine	48.22%	49.86%	45.97%	50.69%
Short-term hospital	1.00%	0.9%	1.11%	0.85%
Home health care	33.88%	31.75%	34.14%	31.37%
Other	16.9%	17.49%	18.78%	17.09%
**Control/ownership of the hospital**				
Government, nonfederal	11.84%	10.78%	11.1%	10.82%
Private, non-profit	73.33%	75.58%	73.95%	75.75%
Private, invest-own	14.83%	13.64%	14.95%	13.43%
**Comorbidities**				
Drug_Abuse	9.91%	4.80%	7.56%	4.70%
Lung_Chronic	45.60%	39.40%	44.93%	38.70%
Diab_CX	42.80%	37.10%	42.13%	36.50%
Dementia	5.30%	7.90%	6.65%	7.90%
Alcohol	5.36%	4.08%	NA	NA
HTN_UNCX	4.16%	5.29%	4.37%	5.40%
Depress	NA	NA	13.04%	11.82%
**Diagnosis codes**				
DXCCSR_MBD021	7.91%	3.43%	5.60%	3.37%
DXCCSR_GEN003	59.58%	51.26%	57.50%	50.57%
DXCCSR_CIR005	42.50%	35%	NA	NA
DXCCSR_RSP008	40.85%	34.53%	40.32%	33.73%
DXCCSR_MBD024	19%	14.63%	NA	NA
DXCCSR_END003	42.85%	37.20%	42.17%	36.54%
DXCCSR_GEN002	41.49%	36.02%	40.64%	35.43%
DXCCSR_FAC010	2.71%	4.69%	2.79%	4.96%
DXCCSR_NVS011	6.13%	8.86%	NA	NA
DXCCSR_MBD001	2.61%	1.29%	2.15%	1.22%
DXCCSR_BLD003	NA	NA	28.30%	23.36%
DXCCSR_END005	NA	NA	51.10%	46.78%
DXCCSR_CIR011	NA	NA	57.62%	54.08%

The most discriminatory continuous variables identified by the logistic regression model (*p*-value < 0.01) and the chi-squared test identified the most prominent binary variables associated with readmission. Thirty-one features were selected for the same cause data; however, 30 were selected for the all-cause dataset. A description of all the features and their higher-level category is provided in Table 2. In addition, Table 2 provides a visual representation of the features added to each dataset, as indicated by the results of the conducted statistical tests. For example, both datasets encompassed age and length of hospital stay, whereas comorbidity associated with depression was exclusively present in the all-cause dataset.

**Table 2:** Features included in the model, along with their categories.

**Feature type**	**Feature**	**Definition**	**Dataset**
Demographics	AGE	Patient's age	Same, all
	FEMALE	Patient's gender (binary, “1” is female)	Same, all
	PAY1	Payment method	Same, all
	PL NCHS	Patient's location (based on NCHS urban-rural code)	Same, all
	ZIPINC QRL	Estimated median house income in the patient's zip code	Same, all
	Resident	Patient's local (binary, “1” is the patient comes from the same state as the hospital)	Same, all
Discharge Information	DMONTH	Patient's discharge month	Same, all
	DISPUNIFORM	Disposition of patients	Same, all
	LOS	Length of the hospital stay	Same, all
	TOTCHG	Patient's inpatient total charges	All
	I10_NDX	Number of ICD-10-CM diagnoses on this discharge	Same, all
	I10_NPR	Number of ICD-10-PCS procedures on this discharge	Same
	I10_SERVICELINE	Service line based on ICD-10-CM/PCS codes	Same
	APRDRG_Risk_Mortality	All patient refined DRG: risk of mortality subclass	Same, all
Hospital-related Information	H_CONTRL	Control/ownership of the hospital	Same, all
	HCUP_ED	HCUP indicator of emergency department record	Same, all
Comorbidities	CMR_DRUG_ABUSE	Drug abuse	Same, all
	CMR_LUNG_CHRONIC	Chronic pulmonary disease	Same, all
	CMR_DIAB_CX	Diabetes with chronic complications	Same, all
	CMR_DEMENTIA	Dementia	Same, all
	CMR_ALCOHOL	Alcohol abuse	Same
	CMR_HTN_UNCX	Hypertension, uncomplicated, and complicated	Same, all
	CMR_DEPRESS	Depression	All
Diagnosis-related information	DXCCSR_MBD021	Stimulant-related disorders	Same, all
	DXCCSR_GEN003	Chronic kidney disease	Same, all
	DXCCSR_CIR005	Myocarditis and cardiomyopathy	Same
	DXCCSR_RSP008	Chronic obstructive pulmonary disease and bronchiectasis	Same, all
	DXCCSR_MBD024	Tobacco-related disorders	Same
	DXCCSR_END003	Diabetes mellitus with complication	Same, all
	DXCCSR_GEN002	Acute and unspecified renal failure	Same, all
	DXCCSR_FAC010	Other aftercare encounter	Same, all
	DXCCSR_NVS011	Neurocognitive disorders	Same
	DXCCSR_MBD001	Schizophrenia spectrum and other psychotic disorders	Same, all
	DXCCSR_BLD003	Aplastic anemia	All
	DXCCSR_FAC010	Other aftercare encounter	All
	DXCCSR_MBD021	Stimulant-related disorders	All

### 3.2 Hyperparameters

As described in the methodology, the dataset was divided into three segments: training, validation, and test sets. The training set was employed for model training, the validation set played a pivotal role in determining optimal hyperparameters for each ML method, and the test set was used in evaluating model performance. An in-depth analysis of the validation set led to the identification of the best hyperparameters for logistic regression, with settings such as solver = “lbfgs” and max_iter = 500. Similarly, for the random forest method, a set of key parameters emerged, including n_estimators = 100, max_features = “sqrt,” min_samples_leaf = 5, min_samples_split = 5, and n_jobs = −1.

### 3.3 Feature selection and classification performance

The outcomes from the feature selection procedure and an application of ML methods, are displayed in Table 3. Feature normalization yielded superior outcomes in the case of the LR method; nevertheless, its effectiveness did not transition to the RF method. Hence, the results of LR with normalization and the results of RF without normalization are presented. In a comprehensive assessment, the RF method demonstrated superior performance compared to the LR across both the same-cause and all-cause datasets. Specifically, in the same-cause dataset, RF exhibited an accuracy of 0.633 and an AUC of 0.607, outperforming LR with an accuracy of 0.603 and an AUC of 0.593. Similarly, within the all-cause dataset, the predictive discriminatory capability of RF in forecasting readmission surpassed that of LR, with an AUC of 0.576. Also, the results of utilization imbalanced data overcoming, including under-sampling, over-sampling, and SMOTE techniques, can be seen in Table 3. The detailed performance of the best performing models also has been presented in Appendix A1.

**Table 3:** Performance metrics for each of the ML methods by considering under-sampling, over-sampling, and smote methods, selected features for each of the conditions by order of selection.

			**LR-normalized**	**Feature set**	**No**	**RF-not normalized**	**Feature set**	**No**
Same- cause (30-day)	Under	ACC	0.6	AGE, GEN003, I10_NPR, ZIPINC_QRTL, PL_NCHS, PAY1, DMONTH, END003, MBD021, RSP008, DISPUNIFORM, I10_NDX, MBD001, I10_SERVICELINE, CMR_DIAB_CX, NVS011, LOS, APRDRG_Risk_Mortality, CIR005, H_CONTRL, GEN002, HCUP_ED, CMR_HTN_UNCX, MBD024, FAC010, FEMALE	24	0.605	AGE, PAY1, DISPUNIFORM, GEN003, DMONTH, I10_NPR, I10_NDX, FEMALE, CMR_LUNG_CHRONIC, ZIPINC_QRTL, GEN002, CMR_HTN_UNCX, CMR_DRUG_ABUSE, FAC010, HCUP_ED, CMR_DEMENTIA, RESIDENT, H_CONTRL, CIR005, CMR_DIAB_CX	20
		SEN	0.582			0.609		
		SPE	0.602			0.605		
		AUC	0.592			0.607		
	Over	ACC	0.603	AGE, GEN003, I10_NPR, RSP008, MBD021, PAY1, DMONTH, GEN002, MBD001, APRDRG_Risk_Mortality, MBD024, FAC010, NVS011, DISPUNIFORM, ZIPINC_QRTL, PL_NCHS, I10_SERVICELINE, END003, CIR005, LOS, H_CONTRL, CMR_LUNG_CHRONIC, CMR_DIAB_CX, CMR_ALCOHOL, FEMALE, HCUP_ED, CMR_DRUG_ABUSE	27	0.633	AGE, PAY1, DISPUNIFORM, GEN003, RSP008, I10_SERVICELINE, FAC010, MBD001, MBD021, CMR_ALCOHOL, CMR_DEMENTIA, RESIDENT, NVS011	13
		SEN	0.576			0.511		
		SPE	0.605			0.645		
		AUC	0.591			0.578		
	Smote	ACC	0.599	AGE, GEN003, I10_NPR, RSP008, ZIPINC_QRTL, DMONTH, MBD021, DISPUNIFORM, CIR005, PAY1, PL_NCHS, I10_NDX, I10_SERVICELINE, H_CONTRL, MBD024, END003, CMR_HTN_UNCX, GEN002, LOS, FAC010, CMR_DRUG_ABUSE, MBD001, NVS011, HCUP_ED, CMR_DEMENTIA	25	0.632	AGE, PAY1, DMONTH, GEN003, RESIDENT	5
		SEN	0.585			0.496		
		SPE	0.601			0.645		
		AUC	0.593			0.571		
All- cause (30-day)	Under	ACC	0.577	I10_NDX, FAC010, GEN003, AGE, DISPUNIFORM, RSP008, BLD003, PAY1, ZIPINC_QRTL, CIR011, MBD021, HCUP_ED, DMONTH, TOTCHG, END003, RESIDENT, PL_NCHS, LOS, H_CONTRL, CMR_DEMENTIA, GEN002, END005, MBD001, CMR_HTN_UNCX	24	0.574	I10_NDX, DISPUNIFORM, FAC010, GEN003, PAY1, RSP008, CMR_HTN_UNCX, CMR_DRUG_ABUSE, RESIDENT, MBD021, MBD001, AGE, LOS, APRDRG_Risk_Mortality, H_CONTRL, DMONTH, CMR_DEMENTIA, BLD003, CMR_LUNG_CHRONIC, ZIPINC_QRTL, FEMALE CMR_DEPRESS	22
		SEN	0.573			0.579		
		SPE	0.579			0.572		
		AUC	0.576			0.576		
	Over	ACC	0.576	I10_NDX, FAC010, GEN003, AGE, DISPUNIFORM, RSP008, PAY1, ZIPINC_QRTL, BLD003, MBD021, CIR011, DMONTH, RESIDENT, LOS, TOTCHG, H_CONTRL, MBD001, APRDRG_Risk_Mortality, CMR_DEMENTIA, CMR_HTN_UNCX, HCUP_ED, GEN002, PL_NCHS, END003, CMR_LUNG_CHRONIC	25	0.563	I10_NDX, DISPUNIFORM, FAC010, GEN003, RSP008, CMR_DRUG_ABUSE, RESIDENT, CMR_HTN_UNCX	8
		SEN	0.572			0.563		
		SPE	0.577			0.563		
		AUC	0.575			0.563		
	Smote	ACC	0.572	I10_NDX, FAC010, GEN003, DISPUNIFORM, AGE, RSP008, PAY1, ZIPINC_QRTL, BLD003, CIR011, MBD021, END005, LOS, CMR_LUNG_CHRONIC, RESIDENT, MBD001, DMONTH, HCUP_ED, H_CONTRL, CMR_DRUG_ABUSE, END003, GEN002, CMR_DIAB_CX, CMR_DEMENTIA, CMR_HTN_UNCX	25	0.499	AGE, PAY1	2
		SEN	0.58			0.602		
		SPE	0.569			0.472		
		AUC	0.575			0.537		

### 3.4 Feature reduction and model refinement

**Figure 2:**
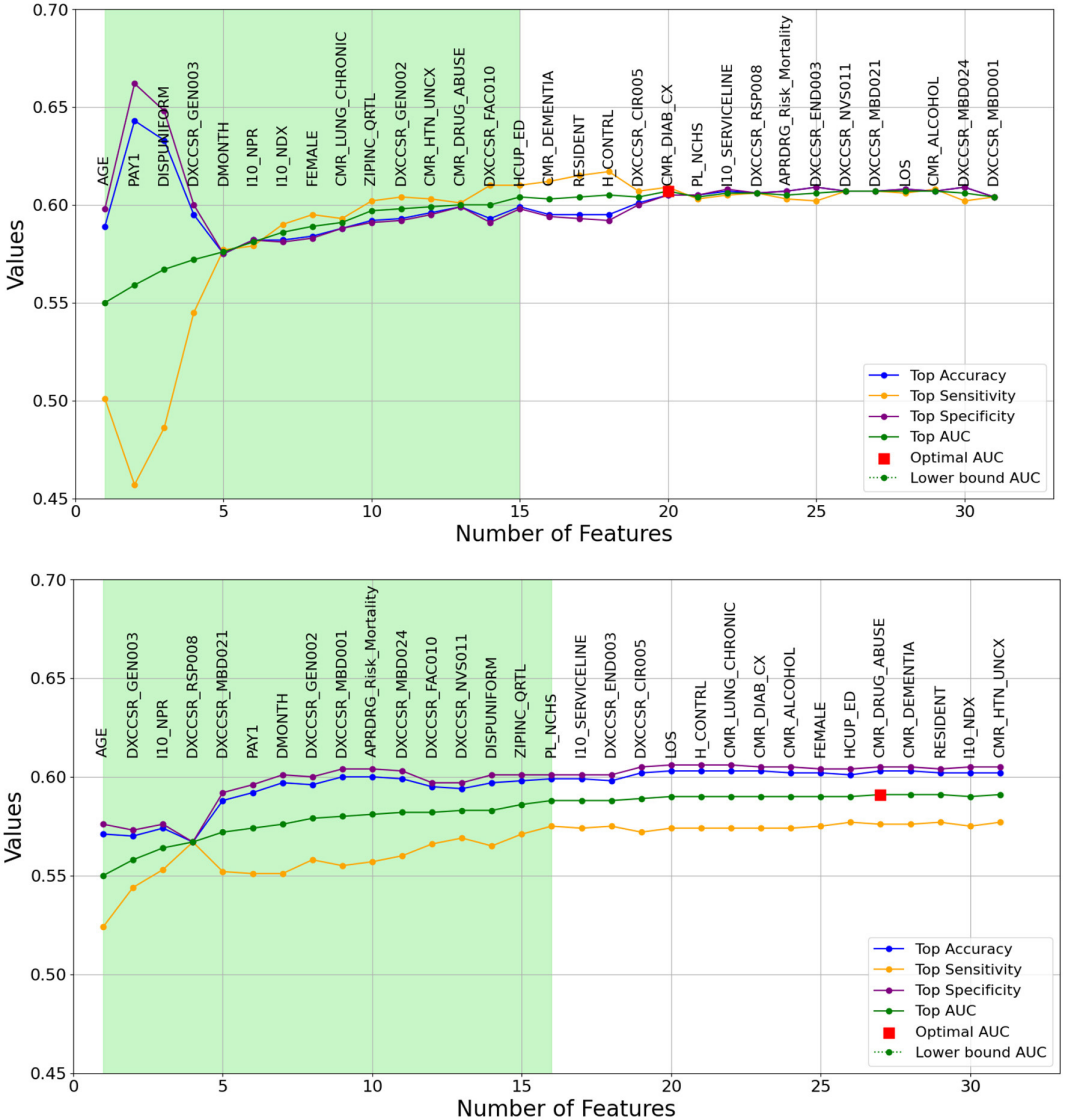
A Graphical representation of feature addition order at each step of the heuristic feature selection process for the optimal 30-day same-cause models. The figure at the **(top)** depicts the random forest model, while the figure at the **(bottom)** represents the logistic regression model. The light green area illustrates the feature set that includes 90% of the AUC range. The X-axis represents the number of features, and the Y-axis represents the AUC values.

**Figure 3:**
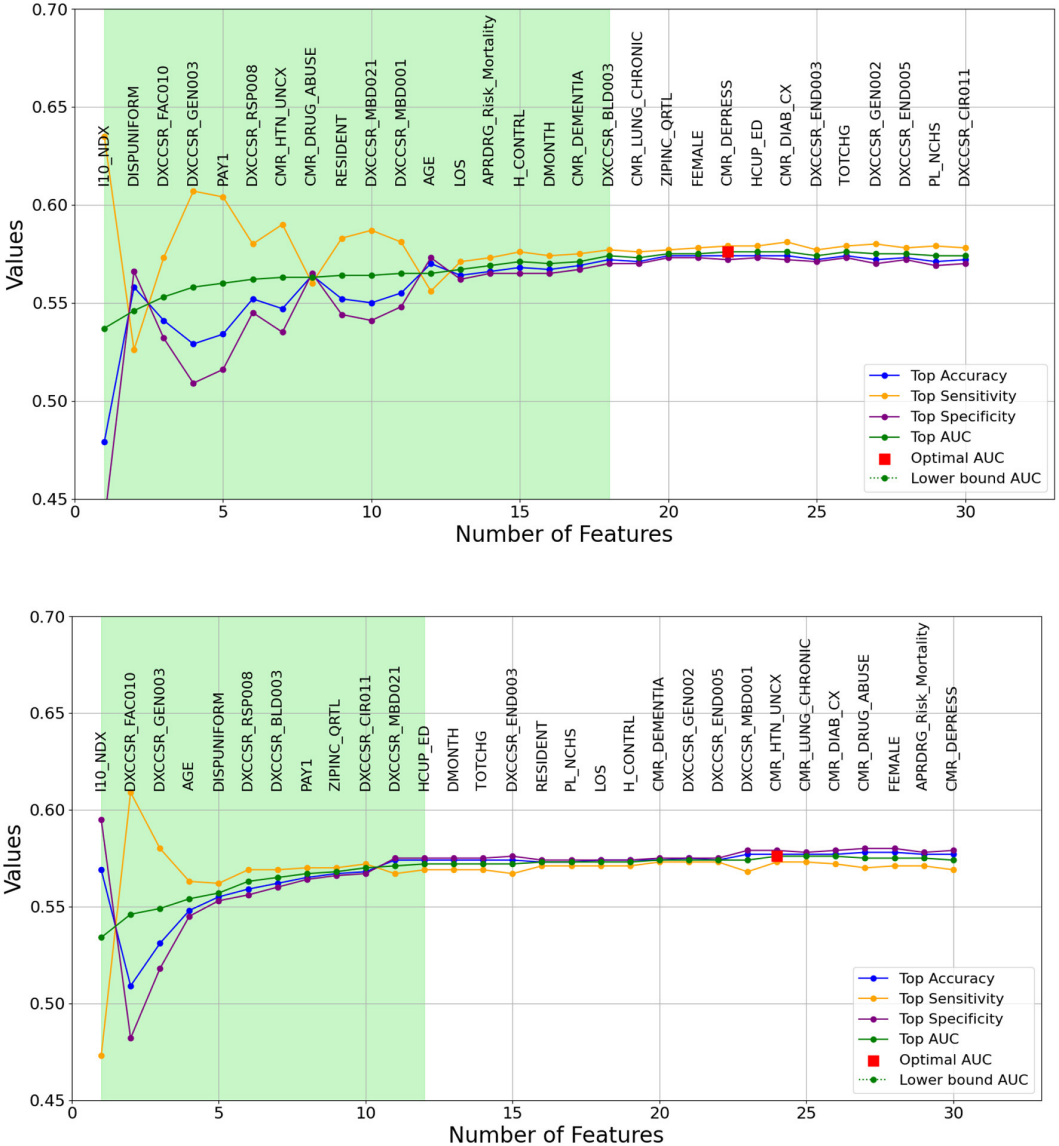
A graphical representation of feature addition order at each step of the heuristic feature selection process for the optimal 30-day all-cause models. The figure at the **(top)** depicts the random forest model, while the figure at the **(bottom)** represents the logistic regression model. The light green area illustrates the feature set that includes 90% of the AUC range. The X-axis represents the number of features, and the Y-axis represents the AUC values.

As previously outlined, the heuristic feature selection process involves incrementally introducing features to the model based on the resulting higher AUC. Figures 2, 3 illustrate the features selected at each step, accompanied by accuracy, sensitivity, specificity, and AUC metrics for each corresponding model. Inference can be drawn that, for the same-cause dataset, the initial steps of feature selection highlighted three pivotal attributes: age, payment method, and chronic kidney disease. In the context of the all-cause dataset, early steps emphasized the significance of variables such as the number of ICD-10-CM diagnoses during discharge, patient disposition, chronic kidney disease, and other post-care encounters, which carry substantial influence on the predictive model. These figures also effectively illustrate the fluctuations observed in each calculated metric. In the early stages, the metrics display significant variation, which gradually diminishes as each feature is progressively integrated into the model. Furthermore, the feature set that encompasses 90% of the AUC range is visually represented by the light green area in Figures 2, 3.

## 4 Discussion

This study leveraged nationwide hospitalization data and machine learning techniques to develop prediction models for 30-day HFR. The results demonstrate the feasibility of achieving reasonable discrimination, with AUC values up to 0.607, for forecasting readmission using a dataset of around 500,000 discharge records. The findings highlight age, payment method, and chronic kidney disease as pivotal predictors of same-cause readmissions. For all-cause readmissions, significant features included the number of diagnoses, disposition status, chronic kidney disease, and post-care encounters. While model discrimination was comparable to other studies, the scale of the dataset and inclusion of diagnosis information provide distinctive value. This research contributes meaningful insights on influential variables and modeling approaches for leveraging expansive datasets to predict readmission and inform care management for heart failure patients.

The discrimination achieved by the RF and LR models developed in this study, with AUC values up to 0.607, are in line with prior analyses that attained AUCs in the range of 0.6–0.66 for predicting HFR (e.g., Philbin and DiSalvo, 1999; Awan et al., 2019a,b). Likewise, the readmission models for other medical settings (e.g., ICU readmission prediction models) have roughly similar range of AUC (Fialho et al., 2012; Viegas et al., 2017). The scale of the dataset likely contributed to the ability to reach this level of performance. However, it is notable that despite a nationwide sample exceeding 480,000 records, discrimination did not substantially surpass results from studies using under 2,000 patients. This suggests inherent challenges in forecasting readmission that persist even with expansive data. The complexity of factors influencing outcomes for heart failure patients impedes predictive modeling. Computational enhancements and more robust feature selection could help maximize the value of large datasets for enhancing model performance. Overall, while the discrimination achieved is on par with past analyses, the outcomes underscore the need to refine predictive modeling methodologies for HFR using big data resources.

The significant predictors identified through the feature selection process in the top-performing model align with the findings of Awan et al. (2019b), who similarly identified age and chronic kidney disease as important factors. Additionally, the results are consistent with studies by Philbin and DiSalvo (1999) and Shams et al. (2015), which recognized insurance status and age as salient features. For all-cause readmissions, the top model emphasized the number of diagnoses, disposition status, chronic kidney disease, and post-care encounters. These features bear similarity to those deemed impactful in previous studies, including the number of procedures, discharge disposition, and secondary diagnoses (Golas et al., 2018; Sharma et al., 2022; Rahman et al., 2023). However, few studies have pointed to post-care encounters as a significant predictor. The distinctions in key features between this analysis and prior literature may stem from the scale and diversity of the nationwide dataset, allowing for the emergence of previously underrecognized predictors. Overall, while there is overlap with some commonly identified predictors, the findings also highlight new factors and the potential value of large datasets for revealing novel drivers of readmissions. Further validation is warranted to confirm the generalizability of these discharge-related and diagnostic features in predicting heart failure readmissions.

Analyzing medical data presents a distinct challenge due to its sophisticated nature, encompassing multidimensional patient profiles, diverse conditions, and complicated interdependencies (Rehman et al., 2022). When dealing with a large amount of medical data, especially in the context of big data, analyzing such information becomes a significant challenge. This study's dataset is notably extensive, encompassing information from over 500,000 patients. This substantial volume of data adds complexity to the analysis process and the development of predictive models. Consequently, identifying trends among patients within the extensive dataset becomes a challenge, leading to models exhibiting unsatisfactory performance metrics. To further analyze the impact of the large sample size, the dataset was reduced by randomly selecting 5,000 records, ~1% of the full dataset. The best-performing ML methods were applied to this smaller subset, and the result of this analysis can be seen in Table 4. Given the class imbalance issue in the sampled dataset, under-sampling, over-sampling, and SMOTE techniques were employed on this dataset too. Additionally, the data division into training, validation, and test sets maintained a consistent 70:15:15 ratio, aligning with the main dataset description. Surprisingly, sampling just 1% of the records enhanced the predictive performance of the best models by ~10% in some cases, measured by AUC. This suggests that while big data provides extensive information, it may also introduce complexities that constrain predictive modeling.

**Table 4:** Discrimination power of the best-performing ML methods on full-size data and reduced sample data with randomly selected 5,000 records.

	**Method**	**Sample size**	**Accuracy**	**Sensitivity**	**Specificity**	**AUC**
Same-cause (30-day)	LR	489,442	0.6	0.582	0.602	0.592
		5,000	0.67	0.588	0.68	0.634
	RF	489,442	0.605	0.609	0.605	0.607
		5,000	0.757	0.529	0.786	0.658
All-cause (30-day)	LR	489,442	0.577	0.573	0.579	0.576
		5,000	0.66	0.65	0.662	0.656
	RF	489,442	0.574	0.579	0.572	0.576
		5,000	0.71	0.5	0.762	0.631

**Figure 4:**
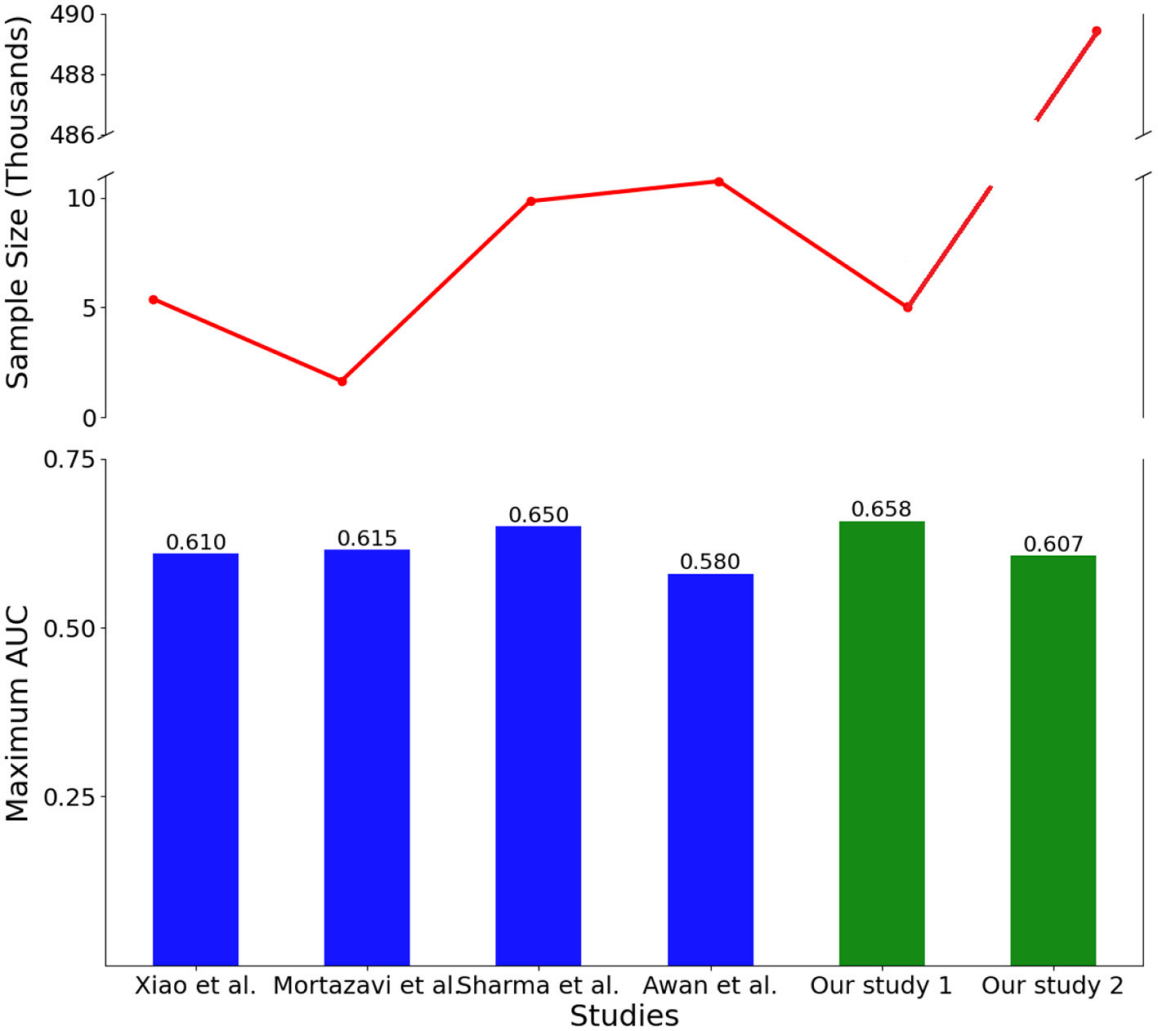
Comparison between sample size and AUC for some of the studies in the literature and our proposed model using both reduced sample size (our study 1) and full sample size (our study 2).

Many past studies analyzing HFR were confined to small datasets, typically comprising just thousands of patients (Zhou et al., 2016). For instance, Mortazavi et al. (2016), Xiao et al. (2018), Awan et al. (2019b), and Sharma et al. (2022) leveraged sample sizes of 5,393, 1,653, 9,845, and 10,757 patients, respectively. The information related to sample sizes and the corresponding AUC values for each of these studies, as well as for our study, encompassing both the full and the reduced sample sizes, can be found in Figure 4. As shown in this Figure, discrimination achieved by these studies topped out at 0.65 AUC, with a sample size of < 11,000 records. Our full sample modeling with around 500,000 records achieved similar discrimination to past literature, reducing the sample to 5,000 records notably improved AUC to 0.658. This indicates that the study introduces a novel modeling approach that achieves robust predictive performance using a large nationwide dataset. This success is attributed to two main factors: a systematic heuristic feature selection technique that identifies important features from high-dimensional data and applying advanced ML algorithms with suitable sampling and normalization techniques. These specialized analytic strategies effectively leverage the big dataset, resulting in strong discrimination for predicting HFR and overcoming inherent complexities.

The light green regions in Figures 2, 3 present the range of features required to maintain 90% of the AUC range. For 30-day same-cause readmission modeling, the full feature set contained 20 variables and achieved an AUC of 0.607 using RF. However, limiting the features to a set of just 15 variables yielded an AUC of 0.601, capturing 90% of the maximal discriminative ability. Similarly, in developing the 30-day all-cause readmission model, the complete feature space had 22 variables, resulting in an AUC of 0.576. Yet, using a reduced feature set of 12 variables could attain an AUC of 0.572, equivalent to 90% of the AUC range. This demonstrates that compromising just 10% of the maximum AUC performance can substantially decrease the number of features from 20 to 15 for same-cause and 24 to 12 for all-cause readmission prediction. The ability to condense the feature space while maintaining most of the predictive power facilitates more efficient modeling. Overall, the light green highlighted regions in the figures provide evidence that nearly the full discriminative capacity can be retained using a parsimonious feature subset, allowing for streamlined model development with minimal impact on predictive performance. Selecting an optimal feature set that balances discrimination and efficiency will be important in translating these models into usable clinical tools.

This study has certain limitations worth acknowledging. First, using a single dataset from the NRD, while providing nationwide representation, constrains generalizability. Validation with external datasets could strengthen reliability. Second, the data lacks detail on outpatient medications, procedures, and healthcare utilization that could provide valuable insights. Incorporating such granular clinical information could enhance predictive modeling. Third, the study was limited to adult patients over 18 years old, and findings may not be generalizable to pediatric populations. Fourth, more complex machine learning methods like neural networks were not explored and could potentially lead to higher predictive performance than the RF and LR models used in this analysis. While this study provides meaningful contributions to predictors and modeling approaches for HFR, the limitations highlight opportunities for additional research to confirm reproducibility, integrate supplementary data sources, expand to broader populations, implement more rigorous validation, and investigate advanced modeling techniques.

## 5 Conclusion

This study aimed to employ ML models utilizing nationwide data to predict readmissions in patients discharged following heart failure. Timely identification of readmission risk among heart failure patients is essential for preventing complications and reducing mortality. Therefore, a heuristic feature selection process, alongside LR and RF methods, was utilized as a key component in the predictive model for readmissions. Our HFR prediction model equips healthcare providers with proactive tools to intervene and reduce emergency hospital readmissions, ultimately leading to improved patient outcomes and reduced healthcare costs. The principal findings of this study are: (1) among the 489,442 patients admitted for HF during 2020, 8.91% were readmitted with the same diagnosis, and 20.65% were readmitted with any diagnosis. (2) using statistical techniques, 31 and 30 features were selected as significant in analyzing readmission for the same-cause and all-cause datasets, respectively. (3) Among the notable features, 10 are linked to diagnosis-related data. Notably, patients readmitted to the hospital displayed significant rates of certain conditions: around 59% had Chronic Kidney Disease, ~40% had Chronic Obstructive Pulmonary Disease and Bronchiectasis, and about 42% had Diabetes Mellitus. (4) Through the application of heuristic feature selection, 20 and 22 features were identified as significant in the HFR prediction model for same-cause and all-cause datasets. (5) The proposed design accurately predicts readmissions for discharged HF patients with an AUC score of 0.607 and 0.576 for same-cause and all-cause datasets, respectively. (6) RF outperformed LR in various scenarios by employing three techniques to counter the imbalanced data challenge. (7) age, payment method, and chronic kidney disease for the same-cause dataset and the number of ICD-10-CM diagnoses, patient disposition, chronic kidney disease, and other post-care encounters for the all-cause dataset are the selected features in the early steps of the feature selection process.

## Data availability statement

Publicly available datasets were analyzed in this study. This data can be found at: https://hcup-us.ahrq.gov/nrdoverview.jsp, Nationwide Readmissions Database (NRD).

## Author contributions

SJ: Conceptualization, Data curation, Formal analysis, Investigation, Methodology, Project administration, Resources, Software, Validation, Visualization, Writing – original draft, Writing – review & editing. MA: Conceptualization, Investigation, Methodology, Project administration, Resources, Supervision, Validation, Visualization, Writing – original draft, Writing – review & editing. NA-F: Conceptualization, Supervision, Writing – review & editing. ER: Supervision, Writing – review & editing.

## Appendix

**Appendix A1:** Detailed performance of the best models.

**Labeling**	**ML model**	**Imbalanced method**	**ACC**	**SEN**	**SPE**	**AUC**	**Pre**	**F1-Score**
Same-cause (30-day)	LR	Over-sampling	0.603	0.576	0.605	0.591	0.593	0.586
	RF	Under-sampling	0.605	0.609	0.605	0.607	0.605	0.609
All-cause (30-day)	LR	Under-sampling	0.577	0.573	0.579	0.576	0.575	0.573
	RF	Under-sampling	0.574	0.579	0.572	0.576	0.572	0.575

## References

[B1] Agency for Healthcare Research and Quality (2020). Introduction to the HCUP Nationwide Readmissions Database (NRD) 2020. Available online at: www.hcup-us.ahrq.gov (accessed February 06, 2024).

[B2] Artetxe A. Beristain A. Grana M. (2018). Predictive models for hospital readmission risk: a systematic review of methods. Comput. Methods Programs Biomed. 164, 49–64. 10.1016/j.cmpb.2018.06.006 30195431

[B3] Ashfaq A. Sant'Anna A. Lingman M. Nowaczyk S. (2019). Readmission prediction using deep learning on electronic health records. J. Biomed. Inform. 97:103256. 10.1016/j.jbi.2019.103256 31351136

[B4] Auffray C. Balling R. Barroso I. Bencze L. Benson M. Bergeron J.. (2016). Making sense of big data in health research: towards an EU action plan. Genome Med. 8, 1–13. 10.1186/s13073-016-0323-y 27338147 PMC4919856

[B5] Awan S. E. Bennamoun M. Sohel F. Sanfilippo F. M. Chow B. J. Dwivedi G.. (2019b). Feature selection and transformation by machine learning reduce variable numbers and improve prediction for heart failure readmission or death. PLoS ONE 14:e0218760. 10.1371/journal.pone.0218760 31242238 PMC6594617

[B6] Awan S. E. Bennamoun M. Sohel F. Sanfilippo F. M. Dwivedi G. (2019a). Machine learning-based prediction of heart failure readmission or death: implications of choosing the right model and the right metrics. ESC Heart Fail. 6, 428–435. 10.1002/ehf2.12419 30810291 PMC6437443

[B7] CDC (2015). International Classification of Diseases, (ICD-10-CM/PCS) Transition – Background. Atlanta, GA: CDC. Available online at: https://www.cdc.gov/nchs/icd/icd10cm_pcs_background.htm (accessed February 06, 2024).

[B8] CMS (2023). Hospital Readmissions Reduction Program (HRRP). Available online at: https://www.cms.gov/ (accessed July 27, 2023).

[B9] Fialho A. S. Cismondi F. Vieira S. M. Reti S. R. Sousa J. M. C. Finkelstein S. N.. (2012). Data mining using clinical physiology at discharge to predict ICU readmissions. Expert Syst. Appl. 39, 13158–13165. 10.1016/j.eswa.2012.05.086

[B10] Golas S. B. Shibahara T. Agboola S. Otaki H. Sato J. Nakae T.. (2018). A machine learning model to predict the risk of 30-day readmissions in patients with heart failure: a retrospective analysis of electronic medical records data. BMC Med. Inform. Decis. Mak. 18, 1–17. 10.1186/s12911-018-0620-z 29929496 PMC6013959

[B11] Guo A. Pasque M. Loh F. Mann D. L. Payne P. R. O. (2020). Heart failure diagnosis, readmission, and mortality prediction using machine learning and artificial intelligence models. Curr Epidemiol Rep. 7, 212–219. 10.1007/s40471-020-00259-w

[B12] Gupta A. Allen L. A. Bhatt D. L. Cox M. DeVore A. D. Heidenreich P. A.. (2018). Association of the hospital readmissions reduction program implementation with readmission and mortality outcomes in heart failure. JAMA Cardiol. 3, 44–53. 10.1001/jamacardio.2017.4265 29128869 PMC5833526

[B13] Jahangiri S. Abdollahi M. Patil R. Rashedi E. Azadeh-Fard N. (2024). An inpatient fall risk assessment tool: Application of machine learning models on intrinsic and extrinsic risk factors. Mach. Learn. Appl. 15:100519. 10.1016/j.mlwa.2023.100519

[B14] Krumholz H. M. Merrill A. R. Schone E. M. Schreiner G. C. Chen J. Bradley E. H.. (2009). Patterns of hospital performance in acute myocardial infarction and heart failure 30-day mortality and readmission. Circ. Cardiovasc. Qual. Outcomes. 2, 407–413. 10.1161/CIRCOUTCOMES.109.883256 20031870

[B15] Low L. L. Lee K. H. Hock Ong M. E. Wang S. Tan S. Y. Thumboo J.. (2015). Predicting 30-day readmissions: performance of the LACE index compared with a regression model among general medicine patients in Singapore. Biomed. Res. Int. 2015:169870. 10.1155/2015/169870 26682212 PMC4670852

[B16] Mortazavi B. J. Downing N. S. Bucholz E. M. Dharmarajan K. Manhapra A. Li S. X.. (2016). Analysis of machine learning techniques for heart failure readmissions. Circ. Cardiovasc. Qual. Outcomes 9, 629–640. 10.1161/CIRCOUTCOMES.116.003039 28263938 PMC5459389

[B17] National Quality Form (2008). National Voluntary Consensus Standards for Hospital Care 2007: Performance Measures. Washington, DC.

[B18] Philbin E. F. DiSalvo T. G. (1999). Prediction of hospital readmission for heart failure: development of a simple risk score based on administrative data. J. Am. Coll. Cardiol. 33, 1560–1566. 10.1016/S0735-1097(99)00059-5 10334424

[B19] Qiu L. Kumar S. Sen A. Sinha A. P. (2022). Impact of the hospital readmission reduction program on hospital readmission and mortality: an economic analysis. Prod. Oper. Manag. 31, 2341–2360. 10.1111/poms.13724

[B20] Rahman M. S. Rahman H. R. Prithula J. Chowdhury M. E. H. Ahmed M. U. Kumar J.. (2023). Heart failure emergency readmission prediction using stacking machine learning model. Diagnostics 13:1948. 10.3390/diagnostics13111948 37296800 PMC10252957

[B21] Rehman A. Naz S. Razzak I. (2022). Leveraging big data analytics in healthcare enhancement: trends, challenges and opportunities. Multimed. Syst. 28, 1339–1371. 10.1007/s00530-020-00736-8

[B22] Ross J. S. Mulvey G. K. Stauffer B. Patlolla V. Bernheim S. M. Keenan P. S.. (2008). Statistical models and patient predictors of readmission for heart failure: a systematic review. Arch. Intern. Med. 168, 1371–1386. 10.1001/archinte.168.13.1371 18625917

[B23] Ru B. Tan X. Liu Y. Kannapur K. Ramanan D. Kessler G.. (2023). Comparison of machine learning algorithms for predicting hospital readmissions and worsening heart failure events in patients with heart failure with reduced ejection fraction: modeling study. JMIR Form Res. 7:e41775. 10.2196/41775 37067873 PMC10152335

[B24] Sarijaloo F. Park J. Zhong X. Wokhlu A. (2021). Predicting 90 day acute heart failure readmission and death using machine learning-supported decision analysis. Clin. Cardiol. 44, 230–237. 10.1002/clc.23532 33355945 PMC7852168

[B25] Savarese G. Lund L. H. (2017). Global public health burden of heart failure. Card. Fail. Rev. 3:7. 10.15420/cfr.2016:25:2 28785469 PMC5494150

[B26] Scholten M. Davidge J. Agvall B. Halling A. (2024). Comorbidities in heart failure patients that predict cardiovascular readmissions within 100 days—an observational study. PLoS ONE 19:e0296527. 10.1371/journal.pone.0296527 38165943 PMC10760770

[B27] Shameer K. Johnson K. W. Yahi A. Miotto R. Li L. I. Ricks D.. (2017). Predictive modeling of hospital readmission rates using electronic medical record-wide machine learning: a case-study using Mount Sinai heart failure cohort. Pac. Symp. Biocomput. 22, 276–287. 10.1142/9789813207813_0027 27896982 PMC5362124

[B28] Shams I. Ajorlou S. Yang K. (2015). A predictive analytics approach to reducing 30-day avoidable readmissions among patients with heart failure, acute myocardial infarction, pneumonia, or COPD. Health Care Manag. Sci. 18, 19–34. 10.1007/s10729-014-9278-y 24792081

[B29] Sharma V. Kulkarni V. McAlister F. Eurich D. Keshwani S. Simpson S. H.. (2022). Predicting 30-day readmissions in patients with heart failure using administrative data: a machine learning approach. J. Card. Fail. 28, 710–722. 10.1016/j.cardfail.2021.12.004 34936894

[B30] Tong R. Zhu Z. Ling J. (2023). Comparison of linear and non-linear machine learning models for time-dependent readmission or mortality prediction among hospitalized heart failure patients. Heliyon 9:e16068. 10.1016/j.heliyon.2023.e16068 37215773 PMC10192765

[B31] Viegas R. Salgado C. M. Curto S. Carvalho J. P. Vieira S. M. Finkelstein S. N.. (2017). Daily prediction of ICU readmissions using feature engineering and ensemble fuzzy modeling. Expert Syst. Appl. 79, 244–253. 10.1016/j.eswa.2017.02.036

[B32] Virani S. S. Alonso A. Aparicio H. J. Benjamin E. J. Bittencourt M. S. Callaway C. W.. (2021). Heart disease and stroke statistics-2021 update: a report from the American Heart Association. Circulation 143, e254–e743. 10.1161/CIR.0000000000000950 33501848 PMC13036842

[B33] Xiao C. Ma T. Dieng A. B. Blei D. M. Wang F. (2018). Readmission prediction via deep contextual embedding of clinical concepts. PLoS ONE 13:e0195024. 10.1371/journal.pone.0195024 29630604 PMC5890980

[B34] Zheng B. Zhang J. Yoon S. W. Lam S. S. Khasawneh M. Poranki S.. (2015). Predictive modeling of hospital readmissions using metaheuristics and data mining. Expert Syst. Appl. 42, 7110–7120. 10.1016/j.eswa.2015.04.066

[B35] Zhou H. Della P. R. Roberts P. Goh L. Dhaliwal S. S. (2016). Utility of models to predict 28-day or 30-day unplanned hospital readmissions: an updated systematic review. BMJ Open 6:e011060. 10.1136/bmjopen-2016-011060 PMC4932323 27354072

